# Supplementary material for: Determination of Complex Formation between Drosophila Nrf2 and GATA4 Factors at Selective Chromatin Loci Demonstrates Transcription Coactivation
Source: Cells. 2023 Mar 19;12(6):938. doi: 10.3390/cells12060938 (PMC10047698; doi:10.3390/cells12060938)
Supplement: Supplementary file 1 [file cells-12-00938-s001.zip › cells-2126003-supplementary.pdf]

Supplementary Material

Determination of Complex Formation between Drosophila Nrf2 and GATA4 Factors at Selective Chromatin Loci Demonstrates Transcription Coactivation

Emma Neidviecky [1], Huai Deng[1,2]

Figure S1

Figure S2

Figure S3

Table S1

Figure S1

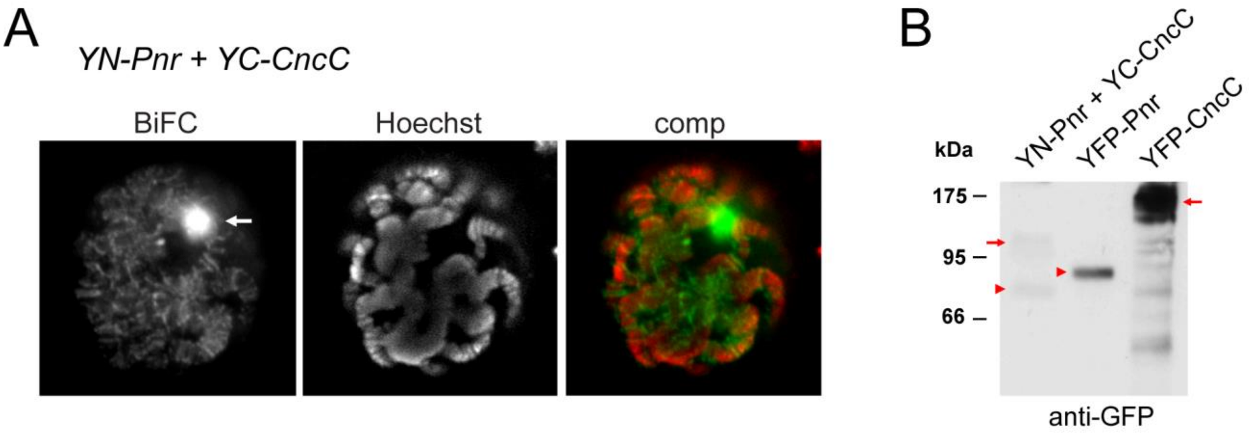

Figure S1. CncC-Pnr BiFC assay in salivary gland cells

(A) CncC-Pnr BiFC assay in salivary gland cells. YN-Pnr and YC-CncC fusion proteins were co-expressed using the *Sgs3-GAL4* driver. Salivary glands were dissected and stained with Hoechst (red). CncC-Pnr BiFC signals (green) were detected both on the polytene chromosome and in a concentrated region (arrow) in the nucleoplasm in less than 10% of cells.

(B) Expression levels of fusion proteins. This is the short exposure result for the western blotting shown in Figure 1C. Extracts of salivary glands that expressed CncC-Pnr BiFC (YN-Pnr + YC-CncC), YFP-CncC, or YFP-Pnr were probed using antibodies against GFP which recognize both the YN and YC fragments of the BiFC fusion proteins. Marker sizes (kDa) are indicated on the left. CncC and Pnr fusion proteins are indicated by arrows and arrowheads, respectively. At higher exposure (Figure 1C), the YFP-CncC band (indicated by the right arrow) becomes indistinguishable from other lower bands.

Figure S2

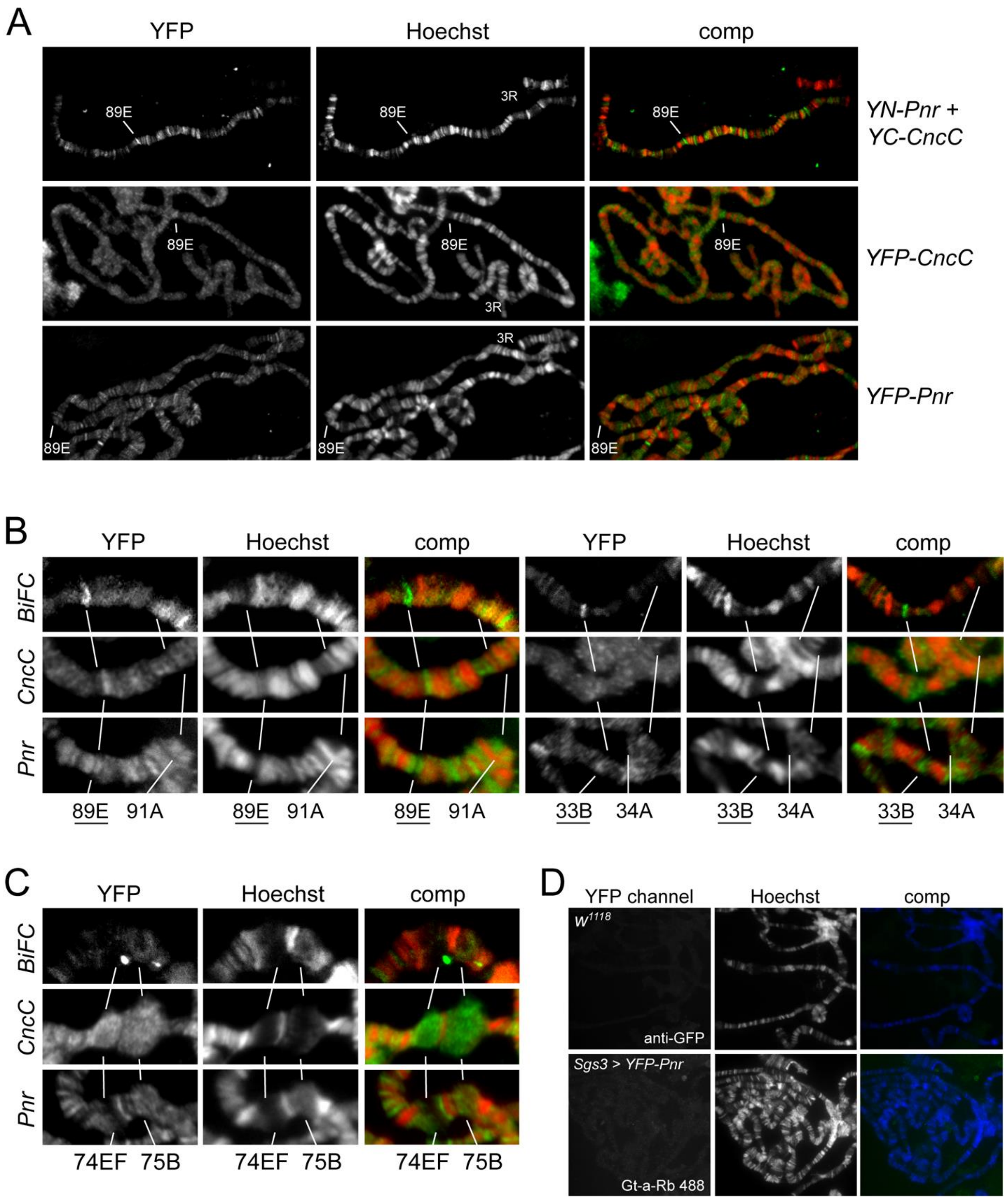
A
YFP
Hoechst
comp
89E
3R
YN-Pnr + YC-CncC
YFP-CncC
YFP-Pnr
B
BiFC
CncC
Pnr
89E
91A
33B
34A
C
74EF
75B
D
YFP channel
w1118
anti-GFP
Sgs3 > YFP-Pnr
Gt-a-Rb 488

Figure S2. Comparison of CncC-Pnr BiFC and individual proteins at chromatin loci

(A) Comparison of the bindings of CncC-Pnr BiFC, YFP-CncC, and YFP-Pnr bindings at the 89E locus. The fusions proteins indicated on the right were expressed in salivary glands using the *Sgs3-GAL4* driver. CncC-Pnr BiFC fluorescence (green) was visualized intrinsically and YFP-CncC and YFP-Pnr were visualized through anti-GFP immunostaining (green). Polytene chromosome spreads were counterstained with Hoechst (red). Chromosome arm 3R and the 89E locus on this chromosome are indicated. Fragments of the 89E region are aligned and compared in Figure 2B.

(B) Comparison of CncC-Pnr BiFC, YFP-CncC, and YFP-Pnr bindings at selected loci. Fragments of polytene chromosome spreads were aligned and compared at loci indicated below. Loci that are highly occupied by CncC-Pnr BiFC complexes are underlined. Adjacent control loci 91A and 34A are highly occupied by YFP-Pnr.

(C) Comparison of CncC-Pnr BiFC, YFP-CncC, and YFP-Pnr bindings at CncC-targeting sites. Intrinsic CncC-Pnr BiFC fluorescence signals (green) were imaged at ecdysone-response puffs (74EF and 75B) that are highly occupied by CncC. YFP-CncC and YFP-Pnr bindings were visualized by anti-GFP immunostaining (green).

(D) Specificity of antibodies for immunostaining. Upper panel: polytene chromosome spreads from wildtype (w^{1118}) salivary glands were stained with anti-GFP followed by Goat anti-rabbit Alexa Fluor 488 secondary antibody. Lower panel: polytene chromosome spreads from salivary glands expressing YFP-Pnr were stained with Goat anti-rabbit Alexa Fluor 488 secondary antibody only. All polytene chromosomes were co-stained using Hoechst (blue). No specific signals were detected in the YFP channel, indicating that the primary and secondary antibodies have no non-specific recognition on the polytene chromosome in our immunofluorescence assays (Figures 2A, 2B, S2A, S2B).

Figure S3

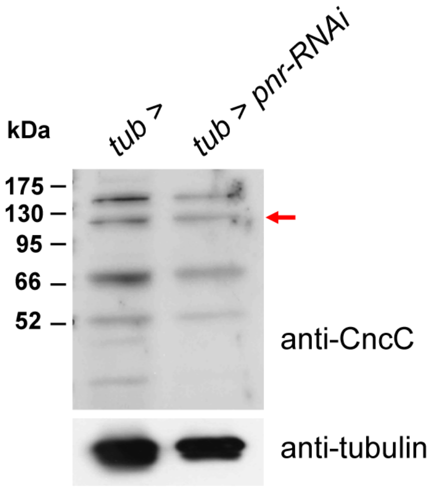

Figure S3. CncC protein levels in the pnr mutant.

Extracts of L2 larvae that express *pnr-RNAi* using *tub-GAL4* (*tub > pnr-RNAi*) and control larvae (*tub-GAL4* only) were analyzed by western bolting using antibodies against CncC and tubulin (loading control). Marker sizes (kDa) are indicated on the left. Endogenous CncC proteins with predicted size are indicated by arrow. Depletion of Pnr by RNAi had no significant effects on the protein level of CncC.

Table S1. Primer sequences used to measure the transcript levels by RT-qPCR

gene	5'-primer	3'-primer
Rp49	CGGATCGATATGCTAAGCTGT	GCGCTTGTTCGATCCGTA
cncC	TGACTCCGCCCAGGATTA	TGTTGTAGCTGAAGTCGTAGGG
pnr	GGAACAGGCTCCACTTTGG	GAGCGAGGGTTTGAGATCAT
Glut3	GACTGATGGTGCGACTCTTG	GCATAGAGCATCGGAACACTT
Actn3	CGAAGAAACAGCCAAAATCG	GGACGCCACTTTGTATTGAAC
Irc	GGGAAATCGAGAGGGTCACT	CGGAATTGGGAAACATCTGA
Pxd	AAGCTCACTGGATTTACTCCTGA	GCACAGCAAACGAGCCATA
Dad	AGCATGTGCCTACGTGACCT	GCCGTTCTCCAGACTCAATG
Ns1	AGGCAAGAGCTCCATCATAAAC	CTCCGCTGGTAAATACGATTCC
dKeap1	CAAGGAGTCGGAGATGTCG	GTAGAGGATGCGTGACATGG
kuk	GTCGATATGGAACCGCTACAA	CTACCACGGAAACTGAAGAGAC
GcklII	ATCTGGAGGAGGCCGAAG	GCGACAGCACCATGATCTC
cal1	AGGAAACACTGGAGGCAATG	CGAACTCGGCCACATCTATTT
cher	CAAGGTGGTGTCCAAGGGTA	TGCCCACGTACAGGATGTT
Or33b	TGCGCAGCACCATGAATA	CCGCAAAGAAGAGAATGTTGAC
Or33c	GAATGGGTCAACTGGGATACTT	TGGAATCGCACCAAGAGTTTA
escl	CAGACCCAGATACCGATGAAAG	TGGTGCTTAACCGGATTGAATA
Ada1-2	GAACTGAATACGGCCAACTTTATG	CAGTCCTTGAGGGTGATTACTG
Ada1-1	GAACTGAATACGGCCAACTTTATG	CAGTCCTTGAGGGTGATTACTG
Nfs1	GACAGGTGCGATTCAACATAAAG	GGAGCATAGCGTCCAATACA
Wdr81	GAACGACTGCATCACTGGAT	GGTGCTGATCCACAAGAGTAAG
dmGlut	TGCTGGTGGTCAAGAAGAAC	CTCCTCGGACCATTCGAAATC
Tom70	CAGTACAAAGTGGAGGCTCTG	AGTTGGGATCAGCATAGTCATTAG
Plzf	CGAAGGAAGCAACGGAGAT	CACTTTGCTGCTGGACTTTAC
Chd1	AAGAGGAGGACTATAGACCCAAG	GTTTGCTGCGAGAGGTAGTT
Cpr	GACCCACTACCTGGAAATTACG	TGGGCGACAGGATTTGATG
CtrlA	GATTTGCAGGCAAGTCACG	CTTCAGTCCCTCGTACATTAGG
Cyp4p1	GCACTCGATGTGGTTCATAGA	GTGTCCACCTCCTCACAAAT
Cyp6v1	GGGCGCCTTATCGCTATTT	GTACAGATCACACACGTCCTTG
Cyt-b5	CGTAACTTCGCTCGAGTTCTTA	TTGTCCTTTCGCTCTCCAC
dl	CGAGCGGTGTTCAGTAACTT	GTGAATCGACCCTTCTGCTC
Egfr	GGATCAGTGCAACGAGGAC	CCTCACATGGACGCACTC
Ets98B	AATCCTTCATCGGAGGCTTG	AGTGCAGCTCCAAATCGG
ewg	TATTCTACGGGACGAGGGAAG	CAAAGGTGGGCAGCAGAT
foxo	AGCAGCAGTCGCCCTAC	CCACGTTGCTGTACGTGTTC
fra	TGTGTGTCCACCATTGAAAGT	AGTGCACTACCGAGCCA
Atx2	GGCAACGGCAATTCTGTG	CTGGTACTGGCGCATCTG
bbc	AATTGTGTGCGGTCTGCT	CATGATGTACACCGATGCGT
bdg	ATCAGGAGGCGGCACAA	CATCGCAACTGTAGCTTTGGT
bsf	TCAGCAGGGAGACATTGAAG	ACAGGAGAGTGGTGTAGGT
Hem	TCCTGGGACCTAAGGCAATA	GCTGCATTACCTGGCTGTAT
Hsc70-3	GCATATTACTGGCCGTCGT	GGTGAAAGCGACGTAGGAG
ifc	TTTGGACACAGCAGACCTATG	CAGGCACACCCACAGAAA
